# Identification and Characterisation of the Gene Cluster Governing Biosynthesis of the Anti‐Mycobacterial Antibiotic Acidomycin

**DOI:** 10.1111/1751-7915.70357

**Published:** 2026-04-26

**Authors:** Anna Vignolle, Martin Zehl, Jaime Felipe Guerrero Garzón, Olha Schneider, Johannes Gafriller, Ulrike Grienke, Rasmus H. Kirkegaard, Sergey B. Zotchev

**Affiliations:** ^1^ Department of Pharmaceutical Sciences, Division of Pharmacognosy University of Vienna Vienna Austria; ^2^ Department of Analytical Chemistry, Faculty of Chemistry University of Vienna Vienna Austria; ^3^ Institute of Science and Technology Austria (ISTA) Klosterneuburg Austria; ^4^ Joint Microbiome Facility, Medical University of Vienna and University of Vienna Vienna Austria; ^5^ Division of Microbial Ecology, Centre for Microbiology and Environmental Systems Science University of Vienna Vienna Austria

**Keywords:** acidomycin, biosynthetic gene cluster, heterologous expression, *Streptomyces*

## Abstract

Acidomycin is an anti‐mycobacterial antibiotic with a unique mode of action, targeting the biotin biosynthesis pathway. Despite being highly active against mycobacteria in vitro, its development as an anti‐tubercular agent has been hindered due to suboptimal pharmacokinetics. Engineering of the acidomycin biosynthesis may yield new analogues with improved pharmacological properties. Here, we describe the identification of the acidomycin biosynthetic gene cluster (BGC) in a *Streptomyces* bacterium isolated from the rhizosphere of Edelweiss. Notably, the acidomycin BGC is located in proximity to the genes for the biosynthesis of stravidins, secondary metabolites targeting a different enzyme in the biotin biosynthesis pathway, and two genes for streptavidins, proteins that strongly bind and sequester biotin. The identity of the acidomycin BGC was confirmed via both gene knock‐out and heterologous expression, which suggested that the fatty acid required for the formation of acidomycin's acyl chain is most likely scavenged from the biotin biosynthesis pathway. CRISPR/Cas9‐assisted knock‐out of the cytochrome P450‐encoding gene in the acidomycin BGC resulted in a significant decrease in its yield but did not abrogate the biosynthesis completely.

## Graphical abstract

Biosynthetic gene cluster for antibiotic acidomycin targeting biotin biosynthesis in mycobacteria was identified in a *Streptomyces* bacterium, and its identity was confirmed by gene knockout and heterologous expression. This cluster is located in proximity to the genes cluster for stravidins targeting a different step in the biotin biosynthesis pathway.

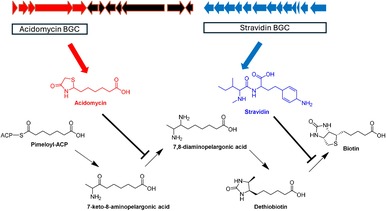

## 1 Introduction

According to the World Health Organization, over 10 million people per year fall ill with tuberculosis (TB) caused by *Mycobacterium tuberculosis* , and up to 1.5 million of those die of the disease (https://www.who.int/health‐topics/tuberculosis#tab=tab_1). Although a number of anti‐mycobacterial drugs are available to treat TB, such as ethambutol, rifampicin and isoniazid (https://www.cdc.gov/tb/treatment/index.html), drug‐resistant TB is on the rise and new anti‐mycobacterial agents are urgently needed. However, the rate of new antibiotic development has been extremely low over the last 20 years mostly due to the frequent re‐discovery of already known antibacterials. At the same time, it might be of interest to reconsider the usefulness of compounds that were discovered in the so‐called ‘Golden Age’ of antibiotics but which development into drugs was abandoned for various reasons. Recent progress in medicinal chemistry as well as advances in understanding the biosynthesis of naturally occurring antibiotics and their biosynthetic engineering may provide new ways for improving drug‐like properties of such ‘forgotten’ antibiotics.

**FIGURE 1:**
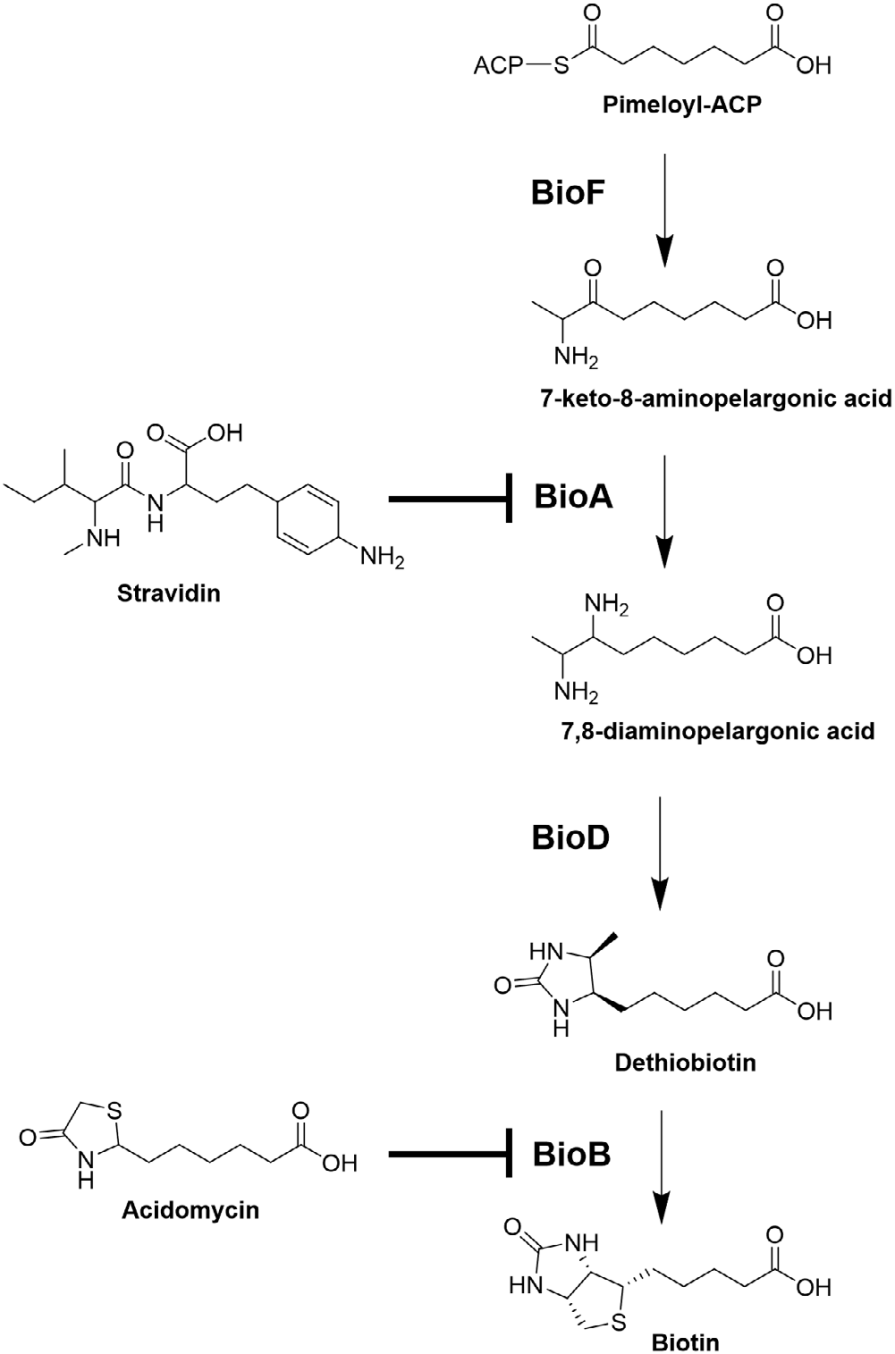
*Streptomyces*‐derived inhibitors of the biotin biosynthesis pathway in bacteria.

Acidomycin (**1**) (Figure 1), an anti‐mycobacterial antibiotic first discovered in 1952, has been isolated from several strains of Gram‐positive bacteria of the order *Streptomyces* (Grundy et al. 1952; Tejera et al. 1952; Umezawa et al. 1953). These bacteria are well known as prolific producers of chemically diverse secondary metabolites with various biological activities, some of which have been developed into clinically used antibiotics (tetracyclines, daptomycin, tobramycin, etc.). Acidomycin, which was alternatively also named mycobacidin and actithiazic acid, belongs to the class of thiazolidin‐4‐one compounds, with a characteristic thiazolidinone ring linked to an aliphatic carboxylic acid side chain at C‐2. It was shown to efficiently and specifically inhibit the growth of *M. tuberculosis* in vitro with MIC values ranging from 0.096 to 6.2 μM, while having almost no effect on other bacteria, fungi or human cells (Bockman et al. 2019). Acidomycin is an antimetabolite that acts as an inhibitor of the biotin synthase BioB, responsible for the conversion of dethiobiotin to biotin (Bockman et al. 2019). Biotin is essential for *M. tuberculosis* due to the dependence of the enzymes involved in mycobacterial cell wall biosynthesis on this co‐factor (Bansal‐Mutalik and Nikaido 2014; Ma et al. 2014). Mycobacteria rely heavily on endogenous biotin production, as they have limited capacity to import biotin from the environment (Lazar et al. 2017). Their thick, lipid‐rich cell envelope reduces uptake of small molecules, including biotin, so when BioB is inhibited, intracellular biotin levels collapse quickly, leading to growth inhibition. Despite being highly active against *M. tuberculosis* in vitro, acidomycin has never been developed into an anti‐mycobacterial drug due to its suboptimal pharmacokinetics, in particular its very short half‐life (Hwang 1952). Interestingly, some *Streptomyces* bacteria were shown to produce stravidins, amiclenomycin‐containing dipeptides that inhibit BioA, the enzyme performing the second step in biotin biosynthesis. These secondary metabolites were shown to act synergistically with the co‐produced biotin‐binding protein streptavidin as antibiotic complex. The structures of acidomycin and stravedin S3 and their targets in the biotin biosynthesis pathway are shown in Figure 1.

Recently, Zhao et al. claimed that the biosynthesis of the 4‐thiazolidinone core of mycobacidin (an alternative name for acidomycin) in *Streptomyces virginiae* NRRL ISP‐5094 is accomplished by a single radical S‐adenosylmethionine sulfurtransferase (Zhao et al. 2025). However, no evidence based on gene knock‐out or heterologous expression has been provided. In this work, we describe the identification and analysis of the acidomycin biosynthesis gene cluster (BGC) in a *Streptomyces* sp. isolated from the rhizosphere of Edelweiss, *Leontopodium nivale* subsp. *alpinum* (Cass.) Greuter, syn (Oberhofer et al. 2019). Moreover, we show that the acidomycin BGC is located in the vicinity of the BGC for stravidins, another antimetabolite shown to inhibit BioA, an aminotransferase essential for biotin formation (Mann et al. 2005; Montaser and Kelleher 2020) and demonstrate production of both. These findings represent the first example of co‐localised gene clusters specifying biosynthesis of secondary metabolites inhibiting two different enzymes involved in the formation of biotin that is essential for survival of *M. tuberculosis* .

## 2 Experimental Procedures

### 2.1 Strains, Plasmids and Oligonucleotides

The bacterial strains, plasmids and oligonucleotides used in this study are listed in Tables S1–S3.

### 2.2 Strain Growth Conditions

*Streptomyces* sp. RLA021 was grown on CP6 solid medium and in TSB, SM17, PM4‐1 and liquid media. *Streptomyces coelicolor* M1154 was grown on CP6 and SFM, and *Streptomyces albus* Del14 on SFM. Liquid cultures were incubated at 28°C and 200 *rpm*. *E. coli* strains were cultured in Luria‐Bertani (LB) medium at 37°C, with shaking at 200 *rpm* for liquid cultures. *Saccharomyces cerevisiae* BY4742 was grown on solid and in liquid yeast synthetic dropout medium (Y1376, leucine, Sigma‐Aldrich). Media were supplemented with antibiotics as appropriate.

CP6 (corn steep liquor (Sigma) 10 g/L, starch soluble (Sigma) 10 g/L, NaCl 3 g/L, (NH_4_)_2_SO_4_ 3 g/L, CaCO_3_ 3 g/L, agar 20 g/L), TSB (Oxoid Tryptone Soya Broth powder [CM129], 30 g/L); SM17 (glucose 2 g/L, glycerol 40 g/L, soluble starch 2 g/L, soya flour 5 g/L, peptone 5 g/L, yeast extract 5 g/L, NaCl 5 g/L and CaCO_3_ 2 g/L); PM4‐1 (glucose 15 g/L, soy meal 15 g/L, corn steep solids (Sigma) 5 g/L, CaCO_3_ 2 g/L and 6 mL/L Trace Elements Solution (mg/mL): FeSO_4_·7H_2_O, 5.0; CuSO_4_·5H_2_O, 0.39; ZnSO_4_·7H_2_O, 0.44; MnSO_4_·H_2_O, 0.15; Na_2_MoO_4_·2H_2_O, 0.01; CoCl_2_·6H_2_O, 0.02; HCl, 50 mL/L); MYM (maltose 4 g/L, yeast extract 4 g/L, malt extract 10 g/L, adjusted to pH 7.3); autoclaved; and supplemented with 2 mL/L trace elements according to the R2YE preparation protocol (Kieser et al. 2000); SFM (soya flour 20 g/L, d‐mannitol 20 g/L).

### 2.3 Isolation and Purification of Acidomycin

Acidomycin was isolated from an upscaled fermentation of *Streptomyces* sp. RLA021 cultivated in MYM medium for 7 days via an optimised procedure based on Miyake et al. (1953). The total fermentation volume was 3 L, distributed across 60 baffled flasks containing 50 mL of culture broth each. Fermentation broth was acidified to pH 2 by the gradual addition of 20% (v/v) sulphuric acid, with pH monitored using indicator paper. The acidified cultures were transferred to 50 mL Falcon tubes and centrifuged for 10 min at 4000 rpm. The resulting supernatants were clarified by filtration and combined into three 1 L portions, each of which was transferred to a separatory funnel. Liquid–liquid extraction was performed using 1 L of *n*‐butyl acetate per portion. The organic phases were collected, and the aqueous phases were extracted a second time with 500 mL of *n*‐butyl acetate. All organic phases were pooled to yield the crude extract, which was subjected to a pre‐purification procedure. The organic phase was washed twice with an equal volume of acidified water (pH 2), during which acidomycin remained in the organic layer. Deprotonation and partitioning into the aqueous phase of acidomycin was achieved by liquid–liquid extraction with an equal volume of aqueous sodium carbonate solution (pH 9).

1. The aqueous phase was washed with 0.6 volumes of *n*‐butyl acetate and
2. Re‐acidified to pH 2 using 20% sulphuric acid.
3. Acidomycin was re‐extracted into *n*‐butyl acetate using equal volume of organic solvent, yielding the pre‐purified extract. The organic phase was then evaporated to dryness under reduced pressure at 40°C using a rotary evaporator. The dry mass of the pre‐purified extract was determined to be 34.5 mg.

The dried extract was finally dissolved in methanol and evaporated to dryness. High‐resolution LC–MS analysis of this crude processed extract showed that the last step has inadvertently led to the near quantitative transformation of free acidomycin to its methyl ester due to the residual sulphuric acid. For the targeted isolation of this compound, 18.0 mg of the processed extract were dissolved in methanol and subjected to flash chromatography using an Interchim PuriFlash 4250 system equipped with PDA, ELSD and a fraction collector. Separation was performed on a PuriFlash C_18_ HQ column (15 μm, 6 g) using a MeOH/H₂O gradient (0 min: 5/95; 30 min: 98/2; 40 min: 98/2). Thirteen fractions (A1–A13) were collected. Fraction A9 contained acidomycin methyl ester (1.46 mg), and its structure was confirmed by HRESIMS as well as 1D and 2D NMR spectroscopy (see Supporting Information).

### 2.4 NMR‐ and LC–MS‐Based Structural Confirmation of Acidomycin and Its Congeners

LC–MS analyses were performed on a Vanquish Horizon UHPLC system (Thermo Fisher Scientific) equipped with an Acquity Premier HSS T3 column, 2.1 × 150 mm, 1.8 μm (Waters) coupled to the ESI source of a timsTOF fleX mass spectrometer (Bruker Daltonics) as described previously (Vignolle et al. 2025).

Structural elucidation of acidomycin methyl ester was performed using one‐ and two‐dimensional NMR spectroscopy. A total of 1.4 mg of the compound was dissolved in deuterated chloroform (CDCl_3_), and spectra were recorded at 298 K.

^1^H and ^13^C NMR spectra (DEPTq), as well as COSY, HSQC and HMBC experiments, were acquired on an Avance NEO 600 NMR spectrometer (Bruker BioSpin) equipped with a Prodigy TCI nitrogen‐cooled cryoprobe with *z*‐gradient. The operating frequencies were 600.18 MHz for ^1^H and 150.92 MHz for ^13^C. Chemical shift values were calibrated to the residual solvent resonances of CDCl_3_ (*δ*_H_ = 7.24 ppm and *δ*_C_ = 77.23 ppm).

High‐resolution LC–MS analysis revealed that the processed methanolic extract was enriched with acidomycin methyl ester. For the targeted isolation of this constituent, 18.0 mg of the processed methanolic extract was dissolved in methanol and subjected to flash chromatography (Interchim PuriFlash 4250 system, Montluçon, France).

This device is equipped with ELSD, PDA and a fraction collector, controlled by Interchim Software. A PuriFlash C_18_ HQ column (15 μm, 6 g) served as the stationary phase. By applying a MeOH/H_2_O gradient (0′ 5%/95%, 30′ 98%/2%, 40′ 98%/2%), 13 fractions (A1 to A13) were obtained. Fraction A9 was identified as acidomycin methyl ester (1.46 mg). Its structure was elucidated by the interpretation of HRESIMS as well as 1D and 2D NMR spectra. The purity of the isolated compound was determined by HRESIMS to be > 90%.

^1^H and ^13^C (DEPTq) 1D as well as COSY, HSQC and HMBC 2D NMR spectra of acidomycin methyl ester (**2**) (1.4 mg) in CDCl_3_ at 298 K were recorded on an Avance NEO 600 NMR spectrometer (Bruker BioSpin) equipped with a N_2_ cryo probe Prodigy TCI with *z*‐gradient (600.18 MHz for ^1^H, 150.92 MHz for ^13^C). Chemical shifts were calibrated using the ^1^H residual solvent signal at *δ* = 7.24 and the ^13^C solvent signal at *δ* = 77.23.

### 2.5 Construction of Knock‐Out Mutants

The integrative shuttle vector pSOK201 (Zotchev et al. 2000), transferable from *E. coli* to *Streptomyces* via conjugation, was used to construct gene knock‐out vectors. DNA fragments homologous to the target genomic region were cloned into pSOK‐201, enabling chromosomal integration via homologous recombination. For gene knock‐out, an internal fragment of the gene of interest was amplified by PCR and inserted into the vector. Introduction of the resulting knock‐out vector into the corresponding *Streptomyces* strain disrupted the open reading frame, generating a loss‐of‐function mutation.

The knock‐out vectors pBGC2.28NRPS, targeting the NRPS gene ctg2_6433, and pBGC2.28PKS, targeting the PKS I gene ctg2_6439 in BGC 2.28 were constructed. For pBGC2.28NRPS, a 1441 bp fragment of the ctg2_6433 gene and for pBGC2.28PKS a 1564 bp fragment of the gene ctg2_6439 were amplified by PCR. Both fragments were cut with the restriction enzymes *Eco*RI and *Hin*dIII and ligated with the 3113 bp fragment of *Eco*RI‐*Hin*dIII cut pSOK201 (Zotchev et al. 2000). The resulting suicide vectors were introduced into *Streptomyces* sp. RLA021 via conjugation from *E. coli* ET12567/pUZ8002 (Kieser et al. 2000).

### 2.6 Generation of Genome Library of *Streptomyces* sp. RLA021

The genome library of *Streptomyces* sp. RLA021 was generated using the CopyControl Fosmid Library Production Kit with pCC1FOS vector (Biosearch Technologies/Epicentre) according to the manufacturer's instructions. The number of fosmid clones required to represent the complete genome was calculated using the formula provided in the kit, resulting in 1152 clones distributed across thirteen 96‐well plates, with each well containing a single *E. coli* EPI300 colony carrying a pCC1FOS vector with a ~40 kb genomic fragment.

The *Streptomyces* sp. RLA021 genome library was screened by pooled PCR of fosmid DNA isolated from *E. coli* EPI300 clones. Screening PCRs were performed on the fosmid DNA using three primer sets targeting the acidomycin BGC: FP1_BGC2.28/RP1_BGC2.28 (829 bp), FP2_BGC2.28/RP2_BGC2.28 (601 bp) and FP3_BGC2.28/RP3_BGC2.28 (518 bp). The fosmid DNA was isolated and the presence of the target BGC was confirmed by sequencing with primer set pCC1‐FP‐seq/pCC1‐RP‐seq (Table S3).

### 2.7 Cloning of the Acidomycin Biosynthesis Gene Cluster

The pYES expression vector used in this work was constructed using the DNA sequence assembly method, utilising the pCGW vector (Xu et al. 2020) as a backbone. The cloning process proceeded as follows: pCGW was digested with *Nde*I/*Pci*I enzymes, yielding three fragments: 181, 2827 and 13,496 bps. To replace the genes for selection of *Saccharomyces cerevisiae* BY4742 clones with the amino acid leucine instead of uracil and tryptophan, the leucine‐carrying vector pCLY10 was digested with *Hpa*I/*Xho*I enzymes, yielding fragments of 3134 and 4857 bps. *S. cerevisiae* BY4742 was employed as a host for the assembly of the pCGW/13496 bps and pCLY10/3134 bps fragments. For the fusion of the two sequences, an SOEing PCR fragment of 1211 bps was generated, comprising two flanks amplified from the pCGW vector (pCLY10‐FP/pCLY10‐RP (484 bps) and pCGW‐FP/pCGW‐RP (747 bps)). The pCLY10‐FP and pCGW‐RP primer set was used for SOEing PCR. The resulting vector, 16,315 bps in size, was treated with *Xho*I to remove a 1075‐bp fragment, which constituted part of the apramycin resistance gene. In the end, the pYES vector (15,240 bps) does not carry the apramycin gene but carries a gene for leucine resistance in yeast.

To generate the pYES‐acidomycin capture vector (pYES‐ACI), a 31 kb BGC 2.28 fragment from fosmid 8A_2F4 was targeted using two flanking homology arms. The complete acidomycin biosynthetic gene cluster was contained on a single fosmid (8A_2F4) in the RLA021 library. A 388 bp left homology arm (LH) was amplified with primers 8A_2F4_LH‐Fw and 8A_2F4_LH‐Rv, and a 389 bp right homology arm (RH) was amplified with primers 8A2F4_RH_FP and 8A2F4_RH_RP. PCRs were performed with Q5 High‐Fidelity 2× Master Mix (New England Biolabs) using gDNA from *Streptomyces* sp. RLA021 as the template.

The pYES vector was digested with *Aat*II and *Sph*I, and the LH (*Aat*II/*Pme*I) and RH (*PmeI*/*Sph*I) fragments were ligated into the backbone using T4 DNA ligase (New England Biolabs). The ligation mixture was transformed into *E. coli* EPI300. The resulting pYES‐ACI vector carrying flanks was then linearised with *Pme*I. The fosmid 8A_2F4 was linearised and both the linearised pYES‐ACI vector and the fosmid were transformed into *Saccharomyces cerevisiae* BY4742 for TAR‐based assembly. Yeast transformation was performed using the lithium acetate/single‐stranded carrier DNA/PEG method (Gietz and Woods 2006). This procedure yielded the acidomycin‐expressing plasmid pYES‐ACI‐BGC2.28, which was transferred to heterologous hosts *Streptomyces albus* Del14 and *Streptomyces coelicolor* M1154 by intergeneric conjugation using *E. coli* ET12567/pUB307 as the helper strain. Introduction of the empty capture vector pYES‐ACI into the same strains served as a control.

### 2.8 CRISPR/Cas9‐Assisted Inactivation of the P450‐Encoding Gene

CRISPR‐cBEST system (Tong et al. 2019) was used to inactivate the P450‐encoding gene ctg2_6435 within the acidomycin gene cluster cloned in *Streptomyces coelicolor* M1154. The identification of protospacers compatible with CRISPR‐cBEST was done using CRISPy‐web (Blin et al. 2016; Table S4). The protospacers were cloned into the linearised pCRISPR‐cBEST plasmid as specified previously (Tong et al. 2019). The resulting constructs were individually introduced in *S. coelicolor* M1154/pYES‐ACI‐BGC2.28 strain via conjugation from *E. coli* ET12567/pUZ8002 (Kieser et al. 2000). Apramycin (50 μg/mL) was used for selection of recombinant *Streptomyces* strains. Primers that amplify several hundred base pairs fragment containing the edited codon were designed (Table S4). Colony PCR was used to amplify the designed DNA fragments directly from the *S. coelicolor* M1154/pYES‐Acidomycin BGC/pCRISPR‐cBEST colonies. Lastly, the PCR products were cleaned up by Monarch^(R)^ PCR and DNA Cleanup Kit (New England Biolabs Inc.) and then sequenced at Microsynth, Austria. pCRISPR‐cBEST plasmid was curated from KO strain after 5 days cultivation at 37°C and 200 *rpm* in YEME medium.

## 3 Results

### 3.1 Identification of the Acidomycin Biosynthetic Gene Cluster

During the LC–MS‐based analysis of the secondary metabolome of a collection of *Streptomyces* spp. from the rhizosphere of Edelweiss (*Leontopodium nivale* subsp. *alpinum*) (Oberhofer et al. 2019), we tentatively identified production of acidomycin by the strain *Streptomyces* sp. RLA021 (Table S6). However, with no MS/MS spectrum of acidomycin available in the literature as reference, it was necessary to isolate the identified compound and confirm its chemical structure as acidomycin with NMR. The aim was to isolate acidomycin from an upscaled fermentation of *Streptomyces* sp. RLA021 in MYM medium using an optimised procedure established by Miyake et al. (T 1953) (see Materials and Methods). Interestingly, during the isolation process, acidomycin was unintentionally converted to its methyl ester, which was finally purified and subjected to 1D and 2D NMR spectroscopy (Figures S1–S9). The structure of the acidomycin methyl ester (**2**) was confirmed by excellent agreement of the acquired spectra with NMR data published previously by Bockman et al. (2019) (Table S7).

From acidomycin's structure it could be assumed that the thiazolidinone moiety might be the product of a non‐ribosomal peptide synthetase (NRPS) cyclisation domain utilising cysteine and activated pimelic acid as precursors.

**FIGURE 2:**
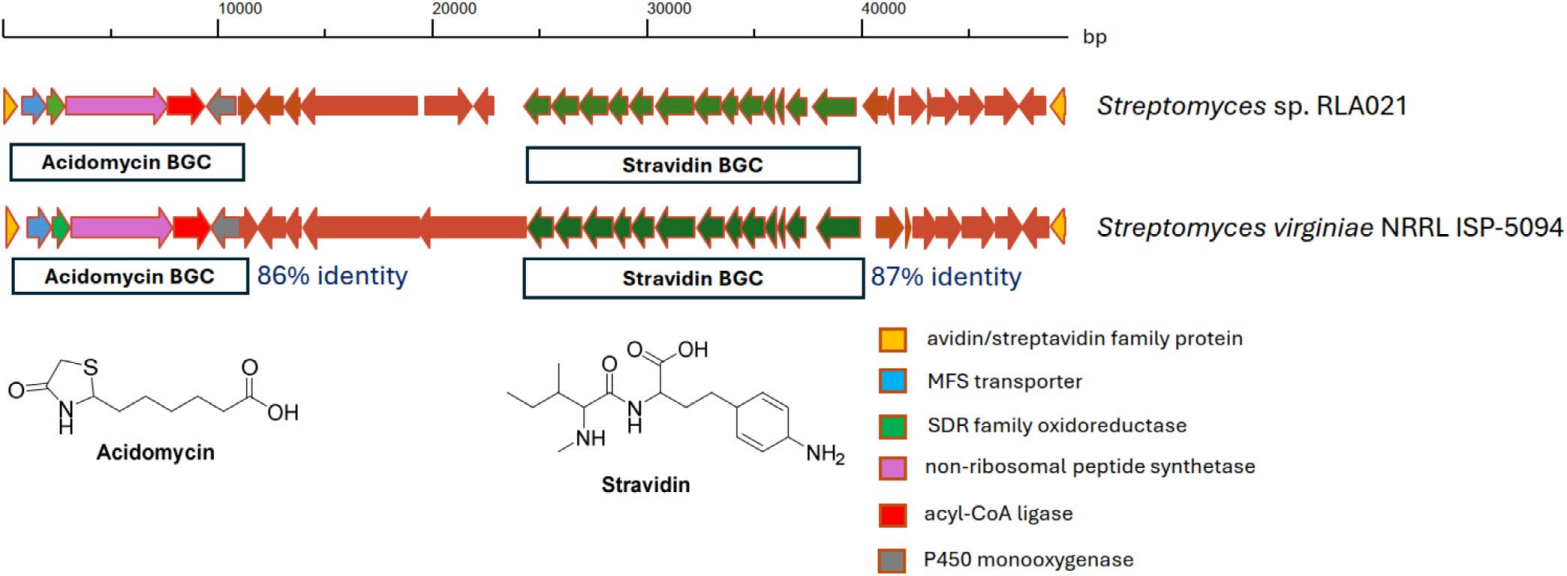
Acidomycin and stravidins biosynthesis gene clusters in *Streptomyces* shown to produce acidomycin. Identity of the acidomycin and stravidin BGCs on nucleotide level is shown.

Analysis of the RLA021 genome with antiSMASH 7.0 (Blin et al. 2023) revealed a hybrid PKSI‐NRPS gene cluster BGC2.28 as a possible candidate for acidomycin BGC since the adenylation domain of NRPS was predicted to be specific for cysteine, and the encoded PKSI might be responsible for the formation of pimelic acid. BGC2.28 (Figure 2) also contains a gene encoding an acyl‐CoA ligase, which may be involved in acidomycin biosynthesis by activating the pimelic acid and loading it to the NRPS. To test this hypothesis, the knock‐out experiments of both the NRPS‐ and PKSI‐encoding genes were carried out (see Section 2). Each gene knock out was accomplished with insertional inactivation using part of the pSOK201 ligated with an internal fragment of the gene of interest.

The pBGC2.28NRPS knock‐out vector was introduced into *Streptomyces* sp. RLA021 via conjugation and three selected transconjugants were cultivated in the liquid media MYM, SM17 and PM4 for 10 days. LC–MS analysis of the methanolic extracts from these cultures revealed complete abrogation of acidomycin production in all three (Figure S10B–D). Apart from acidomycin production, the overall secondary metabolome profiles of the analysed mutants were highly similar to that of the wild‐type strains, indicating minimal off‐target effects. This result established that the NRPS‐encoding gene in BGC 2.28 is essential for the biosynthesis of acidomycin. Interestingly, the inactivation of the mono‐modular PKS Type I‐encoding gene in BGC2.28, presumed to be responsible for the biosynthesis of pimelic acid using a similar strategy (see Section 2), yielded mutants that showed unaltered acidomycin production (Figure S10F–H). Hence, it could be hypothesised that the aliphatic carboxylic acid moiety of acidomycin may be scavenged from primary metabolism, in particular from the biotin biosynthesis pathway. The genes presumed or proven to be involved in the biosynthesis of acidomycin are listed in Table 1.

**TABLE 1:** Annotation of BGC 2.28 of *Streptomyces* sp. RLA021, which encodes the acidomycin biosynthetic pathway.

Gene	Gene product	Putative function
ctg2_6430	Avidin/streptavidin family protein	Biotic sequenstration
ctg2_6431	MFS transporter	Acidomycin efflux
ctg2_6432	SDR family NAD(P)‐dependent oxidoreductase	Unknown
ctg2_6433	Non‐ribosomal peptide synthetase (NRPS)	Formation of the thiazolidinone heterocycle between l‐cystein and pimelic acid
ctg2_6434	AMP‐binding protein	Adenylation of pimelic acid
ctg2_6435	Cytochrome P450	Oxidative decarboxylation?
ctg2_6436	4′‐phosphopantetheinyl transferase superfamily protein	Activation of PCP domain of NRPS
ctg2_6437	MFS transporter	Acidomycin efflux?
ctg2_6438	Thioesterase	Likely not involved in the biosynthesis
ctg2_6439	Type I polyketide synthase	Likely not involved in the biosynthesis

### 3.2 Cloning, Heterologous Expression and Manipulation of the Acidomycin Biosynthesis Gene Cluster

Although the acidomycin‐producing strain *Streptomyces* sp. RLA021 is amenable to genetic manipulation, cloning and expression of the acidomycin BGC in an engineered host with ‘cleaner’ metabolite background would allow more efficient studies on its biosynthesis. A fosmid‐based genome library was constructed for *Streptomyces* sp. RLA021 (see Section 2) and screened utilising pooled PCR and primers designed for the central and flanking regions of the acidomycin BGC (Table S3). This resulted in the identification of a 35.6 kb fragment containing all presumed biosynthetic genes of the acidomycin BGC except the PKS I‐encoding gene, only a small 5′ part of which was present in this single fosmid 8A_2F4. Keeping in mind the fact that inactivation of this gene did not affect acidomycin production in RLA021, we decided to subclone the entire fragment from this fosmid into a vector that can be introduced into *Streptomyces* host.

The 31 kb fragment of the acidomycin BGC contained in the fosmid 8A_2F4 and encompassing genes ctg2_6430 to ctg2_6439 (partially) was excised from the fosmid, purified and incorporated into the pYES vector using transformation‐associated recombination in *Saccharomyces cerevisiae* BY4742 (see Section 2). pYES (Figure S11) is an *S. cerevisiae* — *E. coli* —*Streptomyces* shuttle vector based on pCGW (Xu et al. 2020), which was modified to remove TRP1 and URA3 selection markers, replacing them with LEU2. The yeast colonies obtained after assembly were screened using colony PCR with the same set of primers used for the screening of the genome library. The identified vector pYES‐ACI‐BGC2.28 harbouring acidomycin BGC, was verified for structural integrity by restriction digestion, introduced into *E. coli* TransforMax EPI300 cells and conjugated into two hosts engineered for heterologous expression, *Streptomyces albidoflavus* Del14 (Myronovskyi et al. 2018) and *Streptomyces coelicolor* M1154 (Gomez‐Escribano and Bibb 2011) via tri‐parental conjugation(see Section 2). For each heterologous host, three transconjugants carrying pYES‐ACI‐BGC2.28 were cultivated in GYM fermentation medium for 7 days and methanolic extracts were analysed. The LC–MS data confirmed production of acidomycin by all clones of both hosts, confirming the successful heterologous expression of the identified acidomycin biosynthetic gene cluster (Figure S12). This result also confirmed that the PKS I‐encoding gene is not required for the biosynthesis of the aliphatic side chain of acidomycin.

**FIGURE 3:**
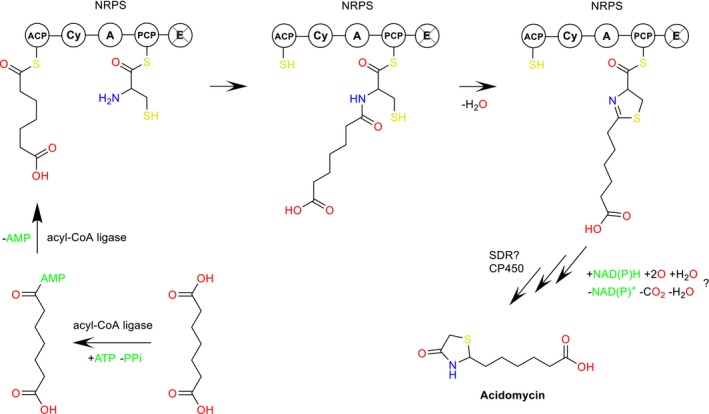
Proposed acidomycin biosynthesis pathway.

We hypothesised that the enzyme cytochrome P450, encoded by the gene ctg2_6435 within the BGC 2.28 might be involved in the oxidative decarboxylation of the thiazoline heterocycle formed from pimelic acid and cysteine by the NRPS encoded by the gene ctg2_6433 (Figure 3). To test this hypothesis, we used the base editing system CRISPR‐cBEST (Tong et al. 2019) for the introduction of a stop codon into the target gene. Two protospacers were designed (Table S4) and cloned in pCRISPR‐cBEST plasmid. The resulting vectors pCRISPR‐cBEST‐C2.28‐6435_A_sp1 and pCRISPR‐cBEST‐C2.28‐6435_B_sp2 were conjugated into *S. coelicolor* M1154/pYES‐ACI‐BGC2.28. Ten transconjugants from each construct were screened by colony PCR followed by sequencing. The sequencing results showed that a stop codon (TAG) was successfully introduced instead of the Gln160 codon (CAG) in four out of 10 clones bearing C2.28_6435_B_sp2 protospacer.

To analyse the effects of the gene ctg2_6435 knock‐out, a fermentation in MYM medium was done with three *S. coelicolor* M1154 clones harbouring the acidomycin BGC with confirmed mutation and *S. coelicolor* M1154 carrying intact acidomycin BGC as a control. The LC–MS analysis of methanolic extracts from these strains showed that the production of acidomycin was significantly diminished but not completely abolished in the knock‐out strains (Figure S13). Moreover, several additional peaks of compounds apparently related to acidomycin were detected in extracts from the mutants. These compounds with the sum formulae C_10_H_17_NO_4_S and C_9_H_17_NO_3_S are hypothesised to be intermediates of the acidomycin biosynthesis (Figure S14).

An abundant congener was detected in the control extract of the strain *S. coelicolor* M1154/pYES‐ACI‐BGC2.28 with an aliphatic side chain that is shorter by a C_2_H_4_ unit and is likely to be derived from glutaric acid instead of pimelic acid (Figure S14). This congener was also detected in the extracts of the knock‐out strains, but at a much lower relative abundance.

### 3.3 Biosynthetic Gene Cluster for Stravidins Is Located Near the Acidomycin BGC in at Least Two Streptomycetes

*S. virginiae* NRRL ISP‐5094 is known as an acidomycin producer, and we readily identified the acidomycin BGC in the genome of this strain (Figure 2). Interestingly, examination of the genes located in the vicinity of the acidomycin BGC in both *Streptomyces* sp. RLA021 and *S. virginiae* NRRL ISP‐5094 led to the identification of the BGC that was shown to govern the biosynthesis of stravidins (Montaser and Kelleher 2020). The latter compounds target the second step in the biotin biosynthesis by inhibiting the BioA enzyme that performs a transamination reaction to convert 7‐keto‐8‐aminopelargonic acid into 7,8‐diaminopelargonic acid using S‐adenosyl‐L‐methionine as the amino donor (Figure 1) (Sirithanakorn and Cronan 2021). Analysis of the methanolic extract from RLA021 indeed showed the presence of stravidins S4 and S5 (Table S5 and Figure S15), thus demonstrating for the first time the ability of a *Streptomyces* bacterium to produce structurally dissimilar compounds that target different steps in the biosynthesis of a cofactor that is essential for the viability of mycobacteria. Production of a mixture of such inhibitors could provide *Streptomyces* an advantage in competing with mycobacteria, which may represent up to 2.9% of the total bacterial population in some soils (Walsh et al. 2019).

Next, we used BLASTP online tool to identify bacteria that may harbour acidomycin BGC using amino acid sequences for NRPS and P450 proteins. This led to the selection of 8 actinomycete strains, namely *Streptomyces amritsarensis* JCM 19660, *Streptomyces avidinii* DSM 40526, *Streptomyces_blastmyceticus* ATCC 23936, *Streptomyces olivoreticuli* DSM 40105, *Streptomyces*_sp._NPDC091204, *Kitasatospora* sp. NPDC056138, *Streptomyces* sp. NPDC007172 and *Streptomyces* sp. NPDC001135. The genomes of these actinomycetes were then queried for the presence of both the complete acidomycin and stravidin BGCs. Only *Streptomyces avidinii, Kitasatospora* sp. NPDC056138 and *Streptomyces* sp. NPDC001135 harboured both BGCs in the same genomic context as RLA021 and *S. virginiae*, while others lacked the stravidin BGC. This may reflect the composition of bacterial community in the samples these actinomycetes were isolated from. Production of a mixture of acidomycin and stravidins could provide *Streptomyces* an advantage in competing with mycobacteria, which may represent up to 2.9% of total bacterial population in some soils (Walsh et al. 2019).

## 4 Discussion

In this study, we report the first experimentally validated identification and characterisation of the acidomycin biosynthetic gene cluster (BGC) and provide insights into its biosynthetic logic, genomic context and evolutionary significance. By combining genome mining, targeted gene inactivation, heterologous expression and CRISPR‐based gene editing, we established a genetic framework for acidomycin biosynthesis and resolved several longstanding uncertainties regarding the origin of its structural features.

A key finding of this work is the demonstration that the NRPS‐encoding gene is essential for acidomycin production, consistent with the hypothesis that cysteine is recruited as the precursor of the thiazolidinone core. In contrast, inactivation of the adjacent type I PKS gene had no effect on acidomycin biosynthesis, both in the native producer and in heterologous hosts. This result strongly suggests that the aliphatic carboxylic acid side chain of acidomycin, apparently derived from pimelic acid, is not synthesised de novo by the BGC‐encoded PKS but is instead scavenged from primary metabolism. Given the structural similarity between pimelic acid and intermediates of the biotin biosynthesis pathway, our data support a model in which acidomycin biosynthesis intersects with biotin metabolism at the level of precursor supply. This metabolic crosstalk mirrors the compound's mode of action as a biotin antimetabolite and further supports the observation that natural product biosynthesis pathways may exploit host primary metabolic fluxes rather than duplicating them.

The successful heterologous expression of the acidomycin BGC further confirmed that the cloned gene set is sufficient for acidomycin biosynthesis and validates the right boundary of the cluster. Importantly, functional expression in the genetically streamlined hosts provides a platform for future pathway engineering and analogue generation, addressing one of the major limitations that historically hindered acidomycin development, namely its unfavourable pharmacokinetics. The detection of naturally occurring congeners with shortened aliphatic side chains further highlights the inherent substrate flexibility of the pathway and suggests that precursor‐directed or enzymatic engineering approaches may expand the chemical diversity of acidomycin‐related metabolites. In particular, the thiazoline ring of acidomycin can be modified by replacing the cysteine‐specific adenylation domain with the one specific for methionine, another sulphur‐containing amino acid. Although rare, such domains exist in both bacterial and fungal NRPS, as recently revealed by Zhang et al. (2025). Another approach would be to engineer the specificity of the acyl‐CoA ligase, which is apparently somewhat promiscuous, towards better affinity for shorter aliphatic chains.

The significant reduction, but not complete loss, of acidomycin production upon inactivation of the cytochrome P450 gene at the right flank of the cluster indicates that this enzyme does not play an essential role in the pathway. We considered it possible that a P450‐encoding gene from the hosts may complement this mutation. While none of the cytochrome P450s encoded in the genomes of *S. coelicolor* or *S. albus* displayed over 32% homology on amino acid sequence level to the protein in question, it is still possible that some of those may assist in the last step of acidomycin biosynthesis. Hence, we propose that acidomycin biosynthesis involves the following steps: activation of pimelic acid by the AMP‐dependent acyl‐CoA ligase, its condensation with l‐cysteine and formation of the thiazoline ring by the NRPS, and finally oxidative decarboxylation to yield the thiazolidinone ring (Figure 3). The last step(s) could be catalysed by the short‐chain dehydrogenase/reductase (SDR), the cytochrome P450 (CP450) or the combined action of both. Members of both enzyme classes were shown to be involved in decarboxylation reactions during natural products biosynthesis (Nguyen et al. 2024). However, detailed mechanistic studies are required to elucidate how exactly the keto group is installed and how the original C=N double bond is removed either by reduction or isomerisation.

Perhaps most striking is the conserved genomic co‐localisation of the acidomycin BGC with the stravidin BGC in two phylogenetically distinct streptomycetes. The ability of a single organism to produce structurally unrelated inhibitors targeting two different enzymes in the biotin biosynthesis pathway represents a rare example of coordinated chemical warfare against a single essential metabolic process. This dual inhibition strategy may provide a strong selective advantage in soil environments where mycobacteria are present, as it reduces the likelihood of resistance development through single‐target escape mutations.

In conclusion, this work establishes the genetic and biochemical foundation for acidomycin biosynthesis, reveals an unexpected integration with biotin metabolism and identifies a unique example of clustered antimetabolite pathways targeting a single essential cofactor. These findings not only advance our understanding of natural product biosynthesis but also reopen acidomycin as a promising scaffold for the development of next‐generation anti‐mycobacterial agents.

## Author Contributions

**Anna Vignolle:** investigation, writing – original draft. **Martin Zehl:** investigation, methodology, validation, writing – original draft, writing – review and editing. **Jaime Felipe Guerrero Garzón:** investigation, methodology, validation, writing – original draft; **Olha Schneider:** investigation, methodology, writing – original draft. **Johannes Gafriller:** investigation, methodology. **Ulrike Grienke:** investigation, writing – original draft, validation, methodology. **Rasmus H. Kirkegaard:** investigation, methodology. **Sergey B. Zotchev:** conceptualization, investigation, funding acquisition, writing – original draft, writing – review and editing, project administration, supervision.

[Corrections added on 13 May 2026, after first online publication: Author Contributions section has been updated in this version.]

## Funding

This work was supported by Universität Wien.

## Conflicts of Interest

The authors declare no conflicts of interest.

## Supporting information


**Table S1:** Bacterial strains used in this work.
**Table S2:** Plasmids used in this work.
**Table S3:** List of primers and oligos.
**Table S4:** List of protospacers used for pCRISPR‐cBEST system.
**Table S5:** BGCs detected in the genome of *Streptomyces* sp. RLA021 with antiSMASH7.0. Potentially unique BGCs are marked in grey.
**Table S6:** Secondary metabolites tentatively identified in different cultures (MYM, SM17, PM4) of *Streptomyces* sp. RLA021 by untargeted LC–MS analysis. Groups of secondary metabolites known or presumed to be biosynthetically related are highlighted by the same colour, whereby usually only the most abundant congeners are reported.
**Figure S1:** Base peak chromatogram (*m/z* 100–2500) obtained by LC–MS in positive ion mode of the isolated acidomycin methyl ester (**2**) subjected to NMR analysis.
**Figure S2:** High resolution ESI‐Qq‐TOF mass spectrum of acidomycin methyl ester (**2**) (A) and simulated isotopic patterns of the [M + H]^+^ ion (B) and [M + Na]^+^ ion (C) of a compound with the sum formula C_10_H_17_NO_3_S.
**Figure S3:** High resolution ESI‐Qq‐TOF MS/MS spectrum of the [M + H]^+^ ion of acidomycin methyl ester (**2**).
**Figure S4:** ^1^H NMR spectrum of acidomycin methyl ester (**2**) in CDCl_3_ at 600 MHz.
**Figure S5:** ^13^C (DEPTq) NMR spectrum of acidomycin methyl ester (**2**) in CDCl_3_ at 151 MHz.
**Figure S6:** COSY spectrum of acidomycin methyl ester (**2**) in CDCl_3_ at 600 MHz.
**Figure S7:** HSQC spectrum of acidomycin methyl ester (**2**) in CDCl_3_ at 600/151 MHz.
**Figure S8:** HMBC spectrum of acidomycin methyl ester (**2**) in CDCl_3_ at 600/151 MHz.
**Figure S9:** Structure of acidomycin methyl ester (**2**) with atom numbering.
**Table S7:** ^1^H (600 MHz, CDCl_3_) and ^13^C NMR data (151 MHz, CDCl_3_) of the isolated acidomycin methyl ester (2) in comparison with literature data (*δ* in ppm).
**Figure S10:** Extracted ion chromatograms (*m/z* 218.0845 ± 0.0011 or *m/z* 218.0845 ± 0.0050) showing the [M + H]^+^ ion of acidomycin obtained by LC–MS in positive ion mode of the MYM culture extracts of the *Streptomyces* sp. RLA021 wild‐type strain (A and E), three BGC2.28 NRPS knock‐out strains (B–D) and three BGC2.28 PKSI knock‐out strains (F–H).
**Figure S11:** Map of the pYES shuttle vector. This vector replicates in *E. coli* , yeast and actinomycetes. Recombinant clones are selectable with chloramphenicol (*cat*) and kanamycin (aph(3′)‐II) in bacteria, and by growth on leucine‐dropout (Leu−) plates in yeast. Replication/partition functions for *E. coli* include *repE* and *sopA*, *sopB* and *sopC*; the vector also carries the incompatibility region of the F plasmid (*incC*). For yeast, it contains the CEN6–ARS4 origin. The *traJ* gene enables conjugative transfer from *E. coli* to actinomycetes, and phi‐C31 encodes the integrase from phage φC31.
**Figure S12:** Extracted ion chromatograms (*m/z* 218.0845 ± 0.0050) showing the [M + H]^+^ ion of acidomycin obtained from the GYM culture extracts of the following strains: *Streptomyces* sp. RLA021 wild‐type (A); *Streptomyces coelicolor* M1154 carrying the ‘empty’ capture vector pYES‐ACI (B, negative control), *S. coelicolor* M1154 carrying the heterologous expression vector pYES‐ACI‐BGC2.28 (C–E, three transconjugants); *Streptomyces albus* Del14 carrying the ‘empty’ capture vector pYES‐ACI (F, negative control); and *S. albus* Del14 carrying the heterologous expression vector pYES‐ACI‐BGC2.28 (G–I, three transconjugants).
**Figure S13:** Extracted ion chromatograms (*m/z* 218.0845 ± 0.0050) showing the [M + H]^+^ ion of acidomycin obtained from the MYM culture extracts of the following strains: *S. coelicolor* M1154 carrying the heterologous expression vector pYES‐ACI‐BGC2.28 (A, positive control); and *S. coelicolor* M1154 carrying the heterologous expression vector pYES‐ACI‐BGC2.28 with knocked‐out cytochrome P450 (B‐D, three mutants).
**Figure S14:** Extracted ion chromatograms obtained from the MYM culture extracts of *S. coelicolor* M1154 carrying the heterologous expression vector pYES‐ACI‐BGC2.28 (A) and *S. coelicolor* M1154 carrying the heterologous expression vector pYES‐ACI‐BGC2.28 with knocked‐out cytochrome P450 (B). The extracted ion chromatograms show the [M + H]^+^ ions of acidomycin (C_9_H_15_NO_3_S, *m/z* 218.0845 ± 0.0050) and compounds with the sum formulae C_9_H_17_NO_3_S (*m/z* 220.1002 ± 0.0050), C_7_H_11_NO_3_S (*m/z* 190.0532 ± 0.0050), C_10_H_17_NO_4_S (*m/z* 248.0951 ± 0.0050) and C_10_H_17_NO_3_S (*m/z* 232.1002 ± 0.0050).
**Figure S15:** High resolution ESI‐Qq‐TOF MS/MS spectra of the [M + H]^+^ ions of stravidin S4 (A) and stravidin S5 (B).

## Data Availability

The genome sequence of *Streptomyces* so. RLA021 has been deposited at NCBI under the BioProject accession number PRJNA1004202, genome submission number SUB15936103.
