# Correction to ‘Identification and Characterisation of the Gene Cluster Governing Biosynthesis of the Anti‐Mycobacterial Antibiotic Acidomycin’

**DOI:** 10.1111/1751-7915.70377

**Published:** 2026-05-20

Vignolle, A., M. Zehl, J. F. G. Garzón, et al. 2026. ‘Identification and Characterisation of the Gene Cluster Governing Biosynthesis of the Anti‐Mycobacterial Antibiotic Acidomycin.’ *Microbial Biotechnology* 19, no. 4: e70357. https://doi.org/10.1111/1751‐7915.70357.

In the originally published version of this article, the Author Contributions section is incomplete. The correct Author Contributions section should be as follows:

## Author Contributions

**Anna Vignolle:** investigation, writing – original draft. **Martin Zehl:** investigation, methodology, validation, writing – original draft, writing – review and editing. **Jaime Felipe Guerrero Garzón:** investigation, methodology, validation, writing – original draft; **Olha Schneider:** investigation, methodology, writing – original draft. **Johannes Gafriller:** investigation, methodology. **Ulrike Grienke:** investigation, writing – original draft, validation, methodology. **Rasmus H. Kirkegaard:** investigation, methodology. **Sergey B. Zotchev:** conceptualization, investigation, funding acquisition, writing – original draft, writing – review and editing, project administration, supervision.

The online version of this article has been corrected accordingly.

