# Supplementary material for: Identification and Characterisation of the Gene Cluster Governing Biosynthesis of the Anti‐Mycobacterial Antibiotic Acidomycin
Source: Microb Biotechnol. 2026 Apr 26;19(4):e70357. doi: 10.1111/1751-7915.70357 (PMC13111796; doi:10.1111/1751-7915.70357)
Supplement: Supplementary file 1 — Table S1: Bacterial strains used in this work. Table S2: Plasmids used in this work. Table S3: List of primers and oligos. Table S4: List of protospacers used for pCRISPR‐cBEST system. Table S5: BGCs detected in the genome of Streptomyces sp. RLA021 with antiSMASH7.0. Potentially unique BGCs are marked in grey. Table S6: Secondary metabolites tentatively identified in different cultures (MYM, SM17, PM4) of Streptomyces sp. RLA021 by untargeted LC–MS analysis. Groups of secondary metabolites known or presumed to be biosynthetically related are highlighted by the same colour, whereby usually only the most abundant congeners are reported. Figure S1: Base peak chromatogram (m/z 100–2500) obtained by LC–MS in positive ion mode of the isolated acidomycin methyl ester (2) subjected to NMR analysis. Figure S2: High resolution ESI‐Qq‐TOF mass spectrum of acidomycin methyl ester (2) (A) and simulated isotopic patterns of the [M + H]+ ion (B) and [M + Na]+ ion (C) of a compound with the sum formula C10H17NO3S. Figure S3: High resolution ESI‐Qq‐TOF MS/MS spectrum of the [M + H]+ ion of acidomycin methyl ester (2). Figure S4: 1H NMR spectrum of acidomycin methyl ester (2) in CDCl3 at 600 MHz. Figure S5: 13C (DEPTq) NMR spectrum of acidomycin methyl ester (2) in CDCl3 at 151 MHz. Figure S6: COSY spectrum of acidomycin methyl ester (2) in CDCl3 at 600 MHz. Figure S7: HSQC spectrum of acidomycin methyl ester (2) in CDCl3 at 600/151 MHz. Figure S8: HMBC spectrum of acidomycin methyl ester (2) in CDCl3 at 600/151 MHz. Figure S9: Structure of acidomycin methyl ester (2) with atom numbering. Table S7: 1H (600 MHz, CDCl3) and 13C NMR data (151 MHz, CDCl3) of the isolated acidomycin methyl ester (2) in comparison with literature data (δ in ppm). Figure S10: Extracted ion chromatograms (m/z 218.0845 ± 0.0011 or m/z 218.0845 ± 0.0050) showing the [M + H]+ ion of acidomycin obtained by LC–MS in positive ion mode of the MYM culture extracts of the Streptomyces sp. RLA021 wild‐type strain (A [file MBT2-19-e70357-s001.docx]

**Identification and characterization of the gene cluster governing biosynthesis of the anti-mycobacterial antibiotic acidomycin**

Anna Vignolle^1^, Martin Zehl^2,3^, Jaime Felipe Guerrero Garzón^1^, Olha Schneider^1^, Johannes Gafriller^1^, Ulrike Grienke^1^, Rasmus H. Kirkegaard^4,5^, Sergey B. Zotchev^1^*

^1^Department of Pharmaceutical Sciences, Division of Pharmacognosy, University of Vienna, 1090 Vienna, Austria.

^2^Department of Analytical Chemistry, Faculty of Chemistry, University of Vienna, 1090 Vienna, Austria.

^3^Institute of Science and Technology Austria (ISTA), Am Campus 1, Klosterneuburg, Austria

^4^Joint Microbiome Facility, Medical University of Vienna and University of Vienna, 1030 Vienna, Austria.

^5^Division of Microbial Ecology, Centre for Microbiology and Environmental Systems Science, University of Vienna, 1090 Vienna, Austria

**Table S1. Bacterial strains used in this work.**

| **Bacterial strain** | **Description** | **Source** |
| --- | --- | --- |
| *Streptomyces* sp. RLA021 | **Wild-type,** acidomycin producer | This work |
| *Streptomyces coelicolor* M1154 | **Host for heterologous expression:** derivate of *Streptomyces coelicolor* M145 [wild-type strain A3(2) lacking plasmids SCP1 and SCP2], ∆*act*; ∆*red*; ∆*cpk*; ∆*cda*; *rpoB*(C1298T); *rpsL*(A262G) | 1 |
| *Streptomyces albus* Del14 | **Host for heterologous expression:** derivative of *Streptomyces albus* J1074 with deletion of 15 gene clusters | 2 |
| *E. coli* DH5α | **General cloning host:** *luxS supE44 ΔlacU169 (ϕ80 lacZΔM15) hsdR17, recA1, endA1, gyrA96, thi-1, relA1* | Thermo Fisher Scientific |
| *E. coli* ET12567/pUZ8002 | **Strain for intergenic conjugation:** RP4 oriT with helper plasmid pUZ8002 (Kan^R^, Cm^R^); methylation deficient (*dam*−, *dcm*−, *hsdM*−) | 3 |
| *E. coli* ET12567 | **Strain for intergenic conjugation:** non-methylating strain without helper plasmid used for tri-parental conjugation: *dam− , dcm− , hsdM−, CmlR* | 4 |
| *E. coli* EPI300-T1R | **Construction of genome library and increased production of fosmids:** *F– mcrA Δ(mrr-hsdRMS-*  *mcrBC) (StrR),*  *φ80dlacZΔM15, ΔlacX74,*  *recA1, endA1, araD139*  *Δ(ara, leu)7697, galU, galK,* *λ– rpsL nupG, trfA, tonA,71*  *dhfr* | Lucigen Corporation |
| *E. coli* ET12567 pUB307 | **Strain with helper plasmid used for tri-parental conjugation***: dam− , dcm− , hsdM−,*  *KanR, CmlR* | 5 |
| One Shot™ Mach1™ T1 Phage-Resistant *Escherichia coli* | **Commercial strain used for cloning:** *F– φ80, lacZΔM15, ΔlacX74, hsdR(rK–, mK+), ΔrecA1398, endA1, tonA* | Thermo Fisher Scientific |
| *Saccharomyces cerevisiae* BY4742 | **Derivative of S288C:** *Mat* α, *his3*Δ1, *leu2*Δ0, *lys2*Δ0, *ura3*Δ0 | 6 |

**Table S2. Plasmids used in this work.**

| **Plasmid** | **Description** | **Source** |
| --- | --- | --- |
| pSOK201 | **Suicide vector used for construction of knock-out vectors:** *NeoR, fd, ColEI ori,*  *oriT, pSG5rep, AmR* | 7 |
| pCGW | **For pYES-generation:** copy number control replicon bearing ‘*oriV-ori2-repE-sopABC’* cassette from pBAC-*lacZ*, inducible with L-arabinose | 8 |
| pCLY10 | **For pYES-generation:** *oriT, attP, VWBint, ori15A, Amp^R^, CEN6-ARS4, LEU2* | 9 |
| pYES | **Shuttle vector, derivate from pCGW:** *LEU2, cat, aph(3’)-II,* | This work |
| pCRISPR-cBEST | **Streptomyces codon optimized spCas9n (D10A)**, modified sgRNA cassette, streptomyces codon optimized rAPOBEC1; *ermE*, tipA* | 10 |

**Table S3: List of primers and oligos**

| **Oligo name** | **Sequence (5’🡪3’)** | **Purpose** |
| --- | --- | --- |
| FP_NRPS_BGC2.28 | GATCAAGCTTCCGACACCACGCTCACCTAC | Amplification of a 1441 bp fragment of the internal region of the NRPS gene in BGC 2.28 of *Streptomyces* sp. RLA021 |
| RP_NRPS_BGC2.28 | GATCGAATTCCCGGTCGAGCTTTCCGTTGG |  |
| FP_T1PKS_BGC2.28 | GATCAAGCTTCTCGATGCTCGCCGTGTCCC | Amplification of a 1578 bp fragment of the internal region of the T1PKS gene in BGC 2.28 of *Streptomyces* sp. RLA021 |
| RP_T1PKS_BGC2.28 | GATCGAATTCACGGCCGACCAGCACGAGAG |  |
| FP1_BGC2.28 | GATCCTGGAAGGCGGTGTAGTTGG | Screening *Streptomyces* sp. RLA021 library for acidomycin BGC, left flanking region |
| RP1_BGC2.28 | CTAGTGATCGCCCTGCTCAACCTC |  |
| FP2_BGC2.28 | GATCGACAAGCCGGACCATGTCAC | Screening *Streptomyces* sp. RLA021 library for acidomycin BGC, central region |
| RP2_BGC2.28 | CTAGAGCGCGTTGAAGTTGTCGTG |  |
| FP3_BGC2.28 | GAGGCGTTGTTGACGAAGCAGTAG | Screening *Streptomyces* sp. RLA021 library for acidomycin BGC, right flanking region |
| RP3_BGC2.28 | CTAGTCCGGTTTCCGCGTCATAGG |  |
| pCC1-FP-seq | GGATGTGCTGCAAGGCGATTAAGTTGG | Screening of fosmid from *Streptomyces* sp. RLA021 library by sequencing |
| pCC1-RP-seq | CTCGTATGTTGTGTGGAATTGTGAGC |  |
| 8A_2F4_LH-Fw | GATCGACGTCGTTGGCGATCAGCTCGATCTG | Construction of capture vector pYES-ACI |
| 8A_2F4_LH-Rv | GATCGTTTAAACGAGCGGATGTACCAGGTGCA |  |
| 8A2F4_RH_FP | GATCGTTTAAACAAGCTGTGCCCGAAGAACACC | Construction of capture vector pYES-ACI |
| 8A2F4_RH_RP | GATCGCATGCAGCATCGGCACGATCTTCGAG |  |
| C2.28_6435_A | CGGTTGGTAGGATCGACGGCTCCTGCAGCGCTTCGGACCGGTTTTAGAGCTAGAAATAGA | ssDNA oligo containing sgRNA for application in CRISPR-cBEST |
| C2.28_6435_B | CGGTTGGTAGGATCGACGGCGATGCAGGCACTCACCCTGGGTTTTAGAGCTAGAAATAGA | ssDNA oligo containing sgRNA for application in CRISPR-cBEST |
| C2.28_6435_C | CGGTTGGTAGGATCGACGGCCAGGGCCCATGCCAGACCCGGTTTTAGAGCTAGAAATAGA | ssDNA oligo containing sgRNA for application in CRISPR-cBEST |
| ctg2_6435_Fw | TCGTACACCACGGGCTTGAG | Screening of KO mutant, ctg2_6435, acidomycin BGC |
| ctg2_6435_Rv | TGGCGGGTTATCAGCAGATG |  |
| CW1:pCRISPR_sgRNA:QC-_fwd | GTACGCGGTCGATCTTGACG | Screening of pCRISPR-cBEST-C2.28-6435 vectors for the presence of designed protospacers |
| pCLY10-FP | ATGGCTTCGGCTGTGATTTC | *leu2*-cassette for pYES vector |
| pCLY10-RP | GTGGGAATACTCAGGTATCG |  |
| pCGW-FP | CGATACCTGAGTATTCCCACCTTGCCCTTGACAGGCATTG | Backbone for pYES vector |
| pCGW-RP | CTCCTGGAGCGACAGTATTG |  |

**Table S4. List of protospacers used for pCRISPR-cBEST system.**

| Name | Sequence (5’-3’) | Target |
| --- | --- | --- |
| C2.28_6435_A_sp1 | CGGTTGGTAGGATCGACGGC**TCCTGCAGCGCTTCGGACCG**GTTTTAGAGCTAGAAATAGA | ctg2_6435, Acidomycin BGC |
| C2.28_6435_B_sp2 | CGGTTGGTAGGATCGACGGC**GATGCAGGCACTCACCCTGG**GTTTTAGAGCTAGAAATAGA | ctg2_6435, Acidomycin BGC |

*Protospacer in bold and light gray background flanked by overhangs compatible with pCRISPR-cBEST.

**Table S5:** BGCs detected in the genome of *Streptomyces* sp. RLA021 with antiSMASH7.0. Potentially unique BGCs are marked in grey.

| **Region** | **No** | **Cluster type** | **Presence in another bacterium** | **Putative product** |
| --- | --- | --- | --- | --- |
| Region 2.1 | 1 | NRPS, NRPS-like, Butyrolactone | antipain biosynthetic gene cluster from *Streptomyces sp.* | antipain |
| Region 2.2 | 2 | NAPAA | ε-Poly-L-lysine biosynthetic gene cluster from *Epichloe festucae* | ε-Poly-L-lysine |
| Region 2.3 | 3 | CDPS | - | cyclodipeptide |
| Region 2.4 | 4 | T3PKS | alkylresorcinol biosynthetic gene cluster from *Streptomyces griseus* subsp. *griseus NBRC 13350* | alkylresorcinol |
| Region 2.5 | 5 | NI-siderophore | *Streptomyces* sp. Sge12 chromosome, complete genome (83% of genes show similarity) | siderophore |
| Region 2.6 | 6 | Melanin | *Streptomyces venezuelae* strain ATCC 21018 (86% of genes show similarity) | melanin |
| Region 2.7 | 7 | Terpene | *Streptomyces* sp. Sge12 chromosome, complete genome (94% of genes show similarity) | terpenoid |
| Region 2.8 | 8 | Terpene | *Streptomyces venezuelae* strain ATCC 21018 (89% of genes show similarity) | terpenoid |
| Region 2.9 | 9 | Lanthipeptide-Class-III | SapB biosynthetic gene cluster from *Streptomyces coelicolor A3(2)* | SapB |
| Region 2.10 | 10 | Terpene | *Streptomyces* sp. 3211 isolate 3 chromosome (94% of genes show similarity) | terpenoid |
| Region 2.11 | 11 | PKS-like, T1PKS | - | unknown |
| Region 2.12 | 12 | NRPS | leupeptin Pr biosynthetic gene cluster from *Streptomyces roseus* | leupeptin |
| Region 2.13 | 13 | T1PKS, hglE-KS | *Streptomyces* sp. Sge12 chromosome (90% of genes show similarity) | polyketide |
| Region 2.14 | 14 | Lanthipeptide-Class-III | - | lanthipeptide |
| Region 2.15 | 15 | Ectoine | ectoine biosynthetic gene cluster from *Streptomyces anulatus* | ectoine |
| Region 2.16 | 16 | Terpene | *Streptomyces* sp. 3211 isolate 3 chromosome (100% of genes show similarity) | terpenoid |
| Region 2.17 | 17 | T1PKS | *Streptomyces* sp. Sge12 chromosome (96% of genes show similarity) | polyketide |
| Region 2.18 | 18 | T1PKS | *Streptomyces* sp. 3211 isolate 3 chromosome (96% of genes show similarity) | polyketide |
| Region 2.19 | 19 | Terpene | geosmin biosynthetic gene cluster from *Streptomyces coelicolor A3(2)* | geosmin |
| Region 2.20 | 20 | RiPP-like | *Streptomyces* sp. Sge12 chromosome (100% of genes show similarity) | modified peptide |
| Region 2.21 | 21 | Thioamitides | - | unknown |
| Region 2.22 | 22 | NRPS, T1PKS, other | *Streptomyces* sp. 3211 isolate 3 chromosome (93% of genes show similarity) | unknown |
| Region 2.23 | 23 | NI-siderophore | *Streptomyces* sp. Sge12 chromosome (100% of genes show similarity) | siderophore |
| Region 2.24 | 24 | Arylpolyene, Lanthipeptide-Class-III | *Streptomyces* sp. INR7 chromosome, complete genome (97% of genes show similarity) | unknown |
| Region 2.25 | 25 | NRPS-like, Butyrolactone | *Streptomyces* sp. Sge12 chromosome (100% of genes show similarity) | unknown |
| Region 2.26 | 26 | T1PKS, Ladderane, Phosphonate | *Streptomyces* sp. Sge12 chromosome (76% of genes show similarity) | unknown |
| Region 2.27 | 27 | NI-siderophore | desferrioxamin B biosynthetic gene cluster from *Streptomyces griseus* subsp. *griseus NBRC 13350* | desferrioxamin B |
| Region 2.28 | 28 | NRPS, T1PKS | Streptomyces sp. Sge12 chromosome (90% of genes show similarity) | unknown |
| Region 2.29 | 29 | Butyrolactone | *Streptomyces* sp. Sge12 chromosome (100% of genes show similarity) | unknown butyrolactone |
| Region 2.30 | 30 | NRPS, T1PKS | coelichelin biosynthetic gene cluster from *Streptomyces coelicolor A3(2)* (72% of genes show similarity) | coelichelin |
| Region 2.31 | 31 | NRPS, NRPS-like | *Streptomyces* sp. Sge12 chromosome (100% of genes show similarity) | tambromycin A, B and C |
| Region 2.32 | 32 | T2PKS | *Streptomyces* sp. 3211 isolate 3 chromosome (94% of genes show similarity) | polyketide |
| Region 2.33 | 33 | Ectoine | ectoine biosynthetic gene cluster from *Streptomyces anulatus* | ectoine |
| Region 2.34 | 34 | NRP-metallophore, NRPS | *Streptomyces venezuelae* strain ATCC 21018 (76% of genes show similarity) | unknown |
| Region 2.35 | 35 | NRPS-like, Terpene | *Streptomyces* sp. Sge12 chromosome (72% of genes show similarity) | unknown |
| Region 2.36 | 36 | Butyrolactone | - | unknown butyrolactone |
| Region 3.1 | 37 | Ectoine | - | ectoine |
| Region 3.2 | 38 | LAP, Thiopeptide | **-** | unknown |

**Table S6:** Secondary metabolites tentatively identified in different cultures (MYM, SM17, PM4) of *Streptomyces* sp. RLA021 by untargeted LC-MS analysis. Groups of secondary metabolites known or presumed to be biosynthetically related are highlighted by the same colour, whereby usually only the most abundant congeners are reported.

**Figure S1.** Base peak chromatogram (*m/z* 100-2500) obtained by LC-MS in positive ion mode of the isolated acidomycin methyl ester (**2**) subjected to NMR analysis.

**Figure S2.** High resolution ESI-Qq-TOF mass spectrum of acidomycin methyl ester (**2**) (A) and simulated isotopic patterns of the [M+H]^+^ ion (B) and [M+Na]^+^ ion (C) of a compound with the sum formula C_10_H_17_NO_3_S.

**Figure S3.** High resolution ESI-Qq-TOF MS/MS spectrum of the [M+H]^+^ ion of acidomycin methyl ester (**2**).


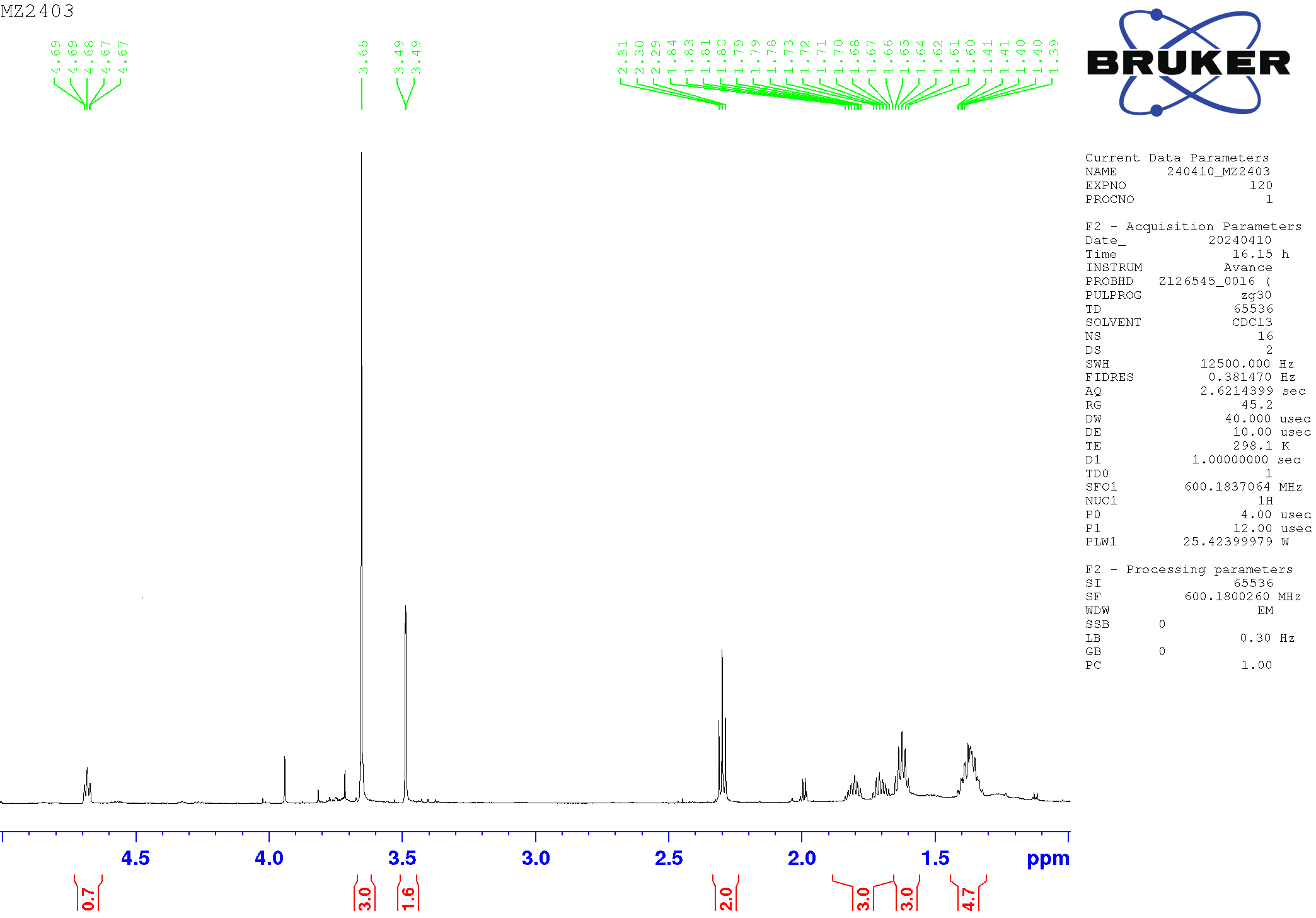


**Figure S4.** ^1^H NMR spectrum of acidomycin methyl ester (**2**) in CDCl_3_ at 600 MHz.


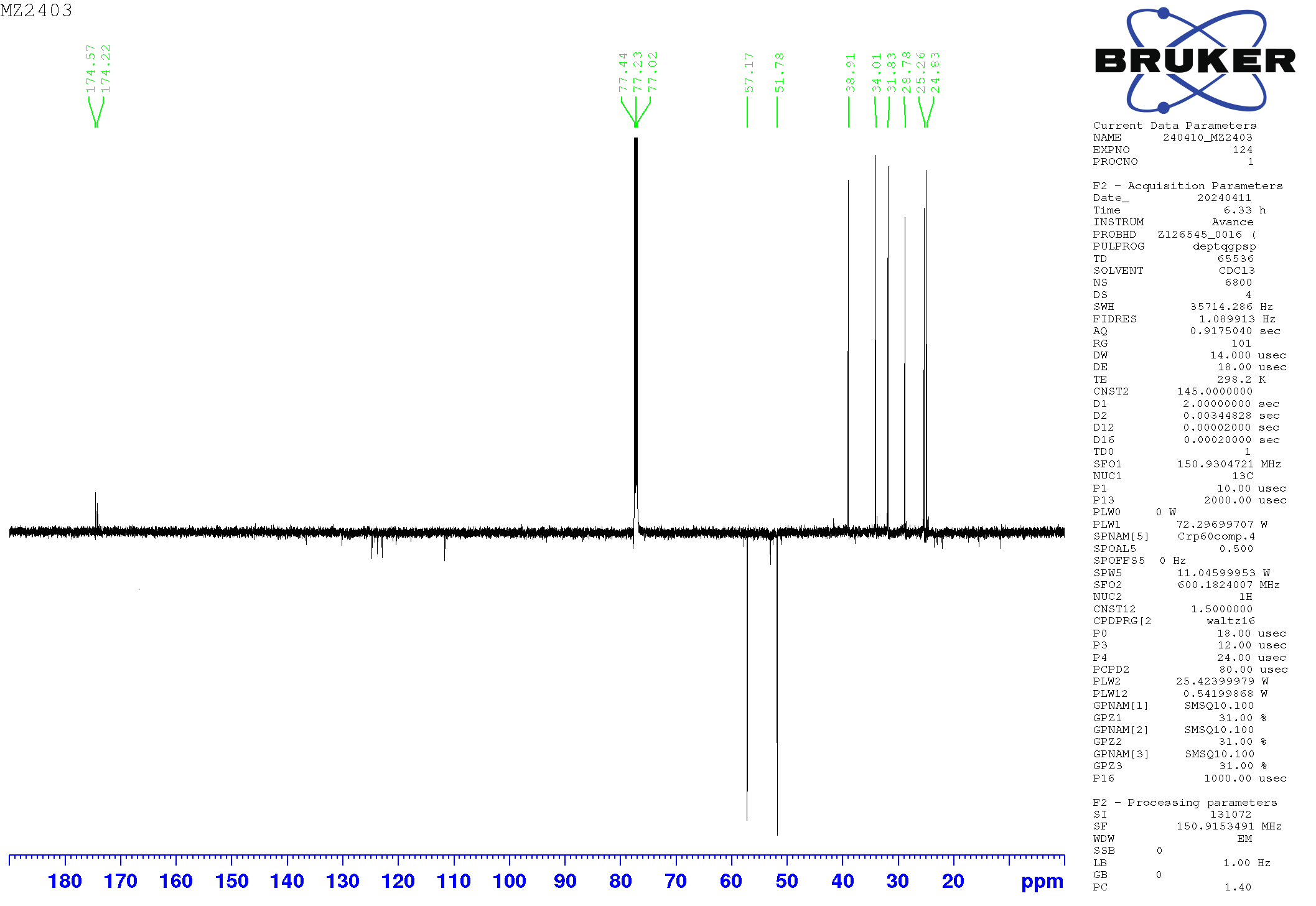


**Figure S5.** ^13^C (DEPTq) NMR spectrum of acidomycin methyl ester (**2**) in CDCl_3_ at 151 MHz.


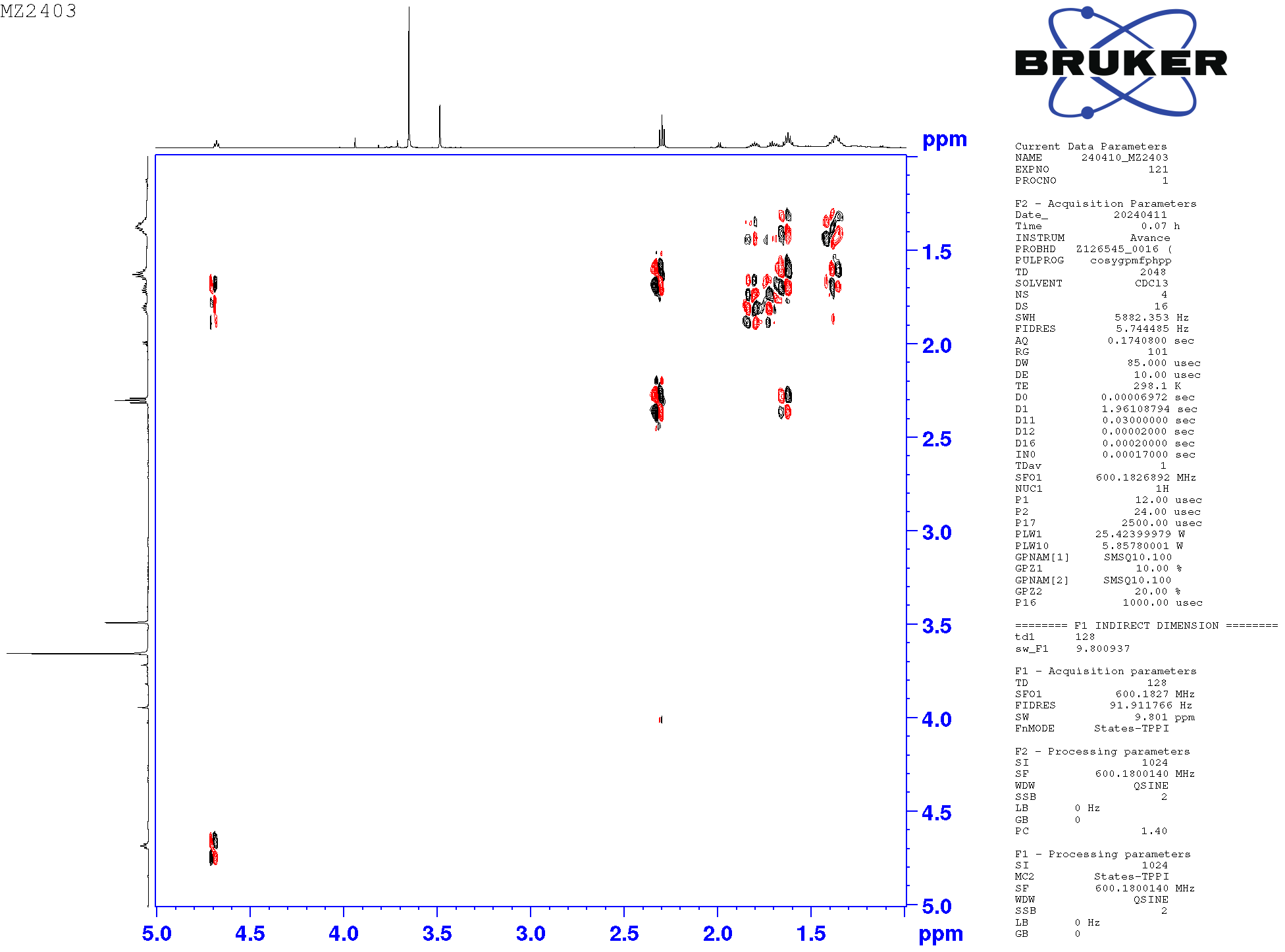


**Figure S6.** COSY spectrum of acidomycin methyl ester (**2**) in CDCl_3_ at 600 MHz.


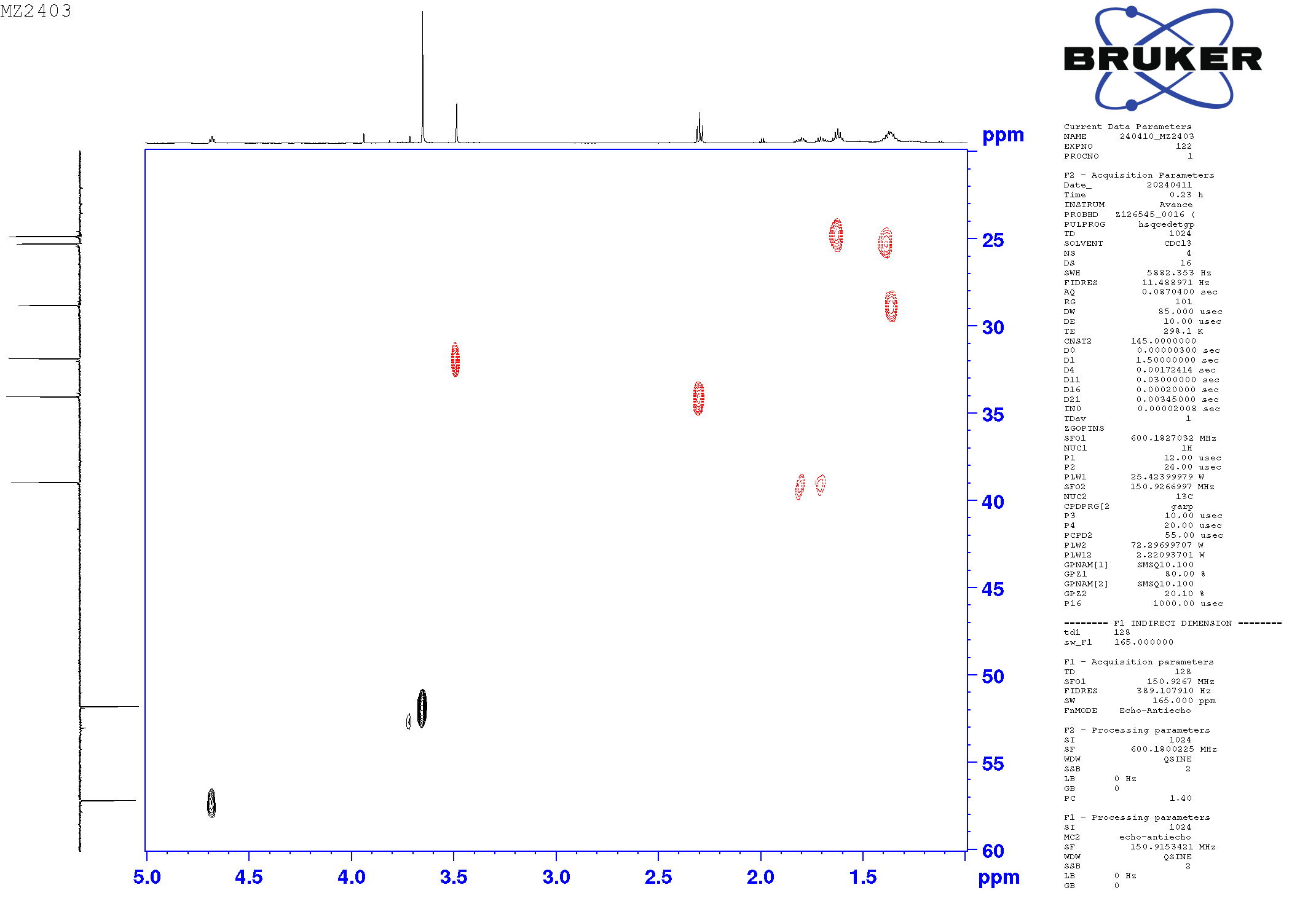


**Figure S7.** HSQC spectrum of acidomycin methyl ester (**2**) in CDCl_3_ at 600/151 MHz.


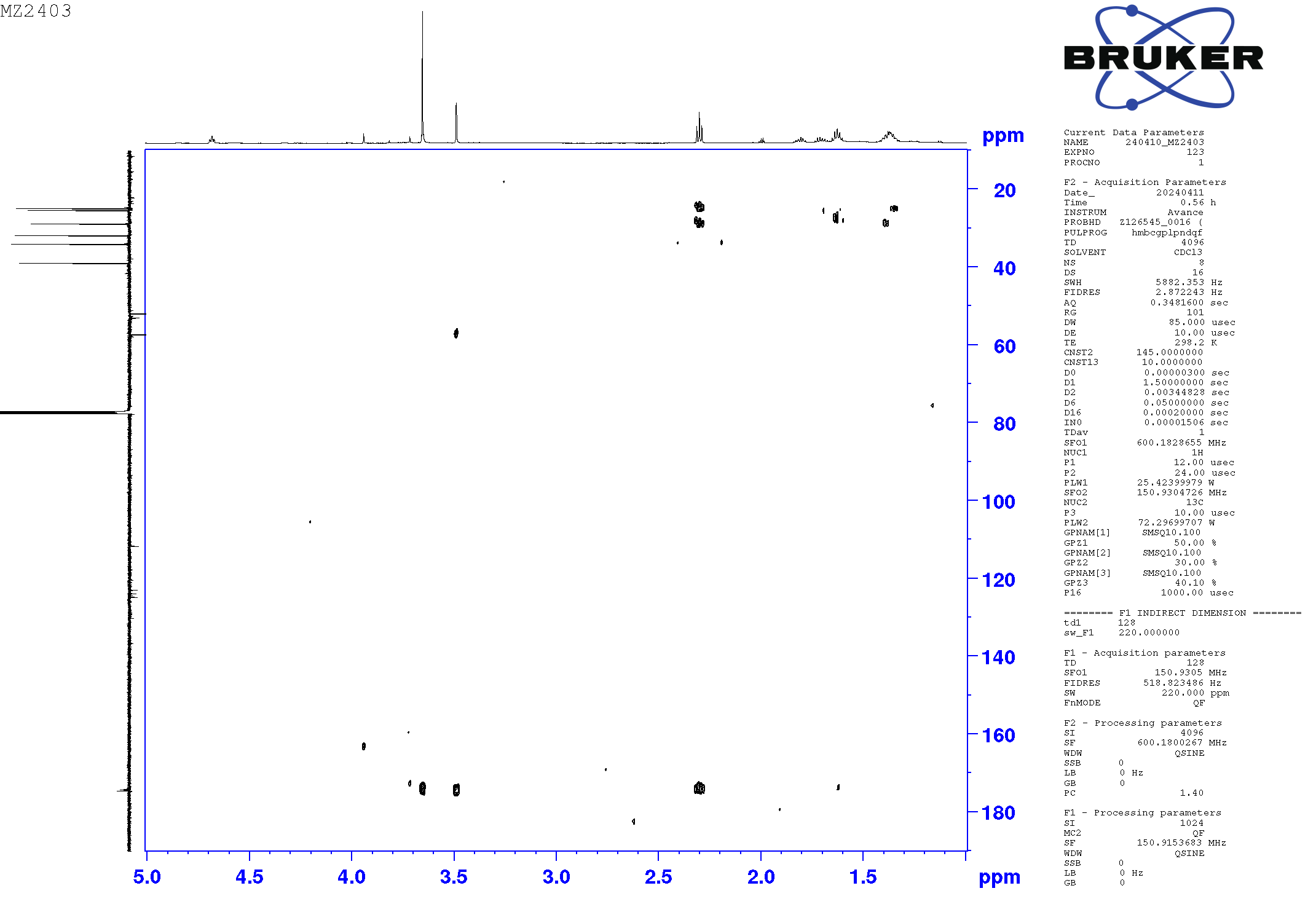


**Figure S8.** HMBC spectrum of acidomycin methyl ester (**2**) in CDCl_3_ at 600/151 MHz.

**Figure S9.** Structure of acidomycin methyl ester (**2**) with atom numbering.

Table S7. ^1^H (600 MHz, CDCl_3_) and ^13^C NMR data (151 MHz, CDCl_3_) of the isolated acidomycin methyl ester (2) in comparison with literature data (*δ* in ppm).

|  | Acidomycin methyl ester (**2**) | | Bockman et al. 2019 | |
| --- | --- | --- | --- | --- |
| Position | δ_H_ (*J* in Hz) | δ_C_, type | δ_H_ (*J* in Hz) | δ_C_, type |
| 1 | — | 174.6, C | — | 174.8, C |
| 2 | 3.49, d (1.1) | 31.8, CH_2_ | 3.50, s | 31.9, CH_2_ |
| 3 | 4.68, td (6.3, 0.9) | 57.2, CH | 4.70, t (6.1) | 57.3, CH |
| 4a | 1.76−1.84, m | 38.9, CH_2_ | 1.76−1.90, m | 38.7, CH_2_ |
| 4b | 1.66−1.74, m |  | 1.68−1.77, m |  |
| 5 | 1.31−1.42, m, ov | 25.3, CH_2_ | 1.24−1.52, m, ov | 25.0, CH_2_ |
| 6 | 1.31−1.42, m, ov | 28.8, CH_2_ | 1.24−1.52, m, ov | 28.6, CH_2_ |
| 7 | 1.62, tt (7.4, 7.4) | 24.8, CH_2_ | 1.63, dt (14.5, 7.6) | 24.6, CH_2_ |
| 8 | 2.30, t (7.4) | 34.0, CH_2_ | 2.30, t (7.2) | 33.8, CH_2_ |
| 9 | — | 174.2, C | — | 174.0, C |
| 10 | 3.65, s | 51.8, CH_3_ | 3.66, s | 51.5, CH_3_ |

**Figure S10.** Extracted ion chromatograms (*m/z* 218.0845 ± 0.0011 or *m/z* 218.0845 ± 0.0050) showing the [M+H]^+^ ion of acidomycin obtained by LC-MS in positive ion mode of the MYM culture extracts of the *Streptomyces* sp. RLA021 wild-type strain (A and E), three BGC2.28 NRPS knock-out strains (B-D), and three BGC2.28 PKSI knock-out strains (F-H).


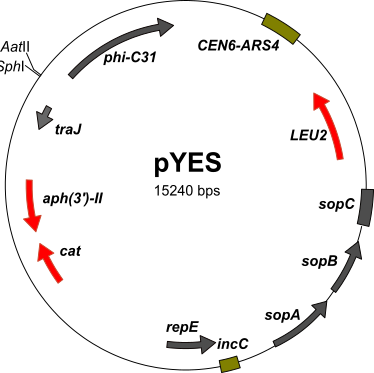


**Figure S11.** Map of the pYES shuttle vector. This vector replicates in *E. coli*, yeast, and actinomycetes. Recombinant clones are selectable with chloramphenicol (*cat*) and kanamycin (aph(3′)-II) in bacteria, and by growth on leucine-dropout (Leu−) plates in yeast. Replication/partition functions for *E. coli* include *repE* and *sopA*, *sopB*, and *sopC*; the vector also carries the incompatibility region of the F plasmid (*incC*). For yeast, it contains the CEN6–ARS4 origin. The *traJ* gene enables conjugative transfer from *E. coli* to actinomycetes, and phi-C31 encodes the integrase from phage φC31.

**Figure S12.** Extracted ion chromatograms (*m/z* 218.0845 ± 0.0050) showing the [M+H]^+^ ion of acidomycin obtained from the GYM culture extracts of the following strains: *Streptomyces* sp. RLA021 wild-type (A); *Streptomyces coelicolor* M1154 carrying the “empty” capture vector pYES-ACI (B, negative control), *S. coelicolor* M1154 carrying the heterologous expression vector pYES-ACI-BGC2.28 (C-E, three transconjugants); *Streptomyces albus* Del14 carrying the “empty” capture vector pYES-ACI (F, negative control); and *S. albus* Del14 carrying the heterologous expression vector pYES-ACI-BGC2.28 (G-I, three transconjugants).

**Figure S13.** Extracted ion chromatograms (*m/z* 218.0845 ± 0.0050) showing the [M+H]^+^ ion of acidomycin obtained from the MYM culture extracts of the following strains: *S. coelicolor* M1154 carrying the heterologous expression vector pYES-ACI-BGC2.28 (A, positive control); and *S. coelicolor* M1154 carrying the heterologous expression vector pYES-ACI-BGC2.28 with knocked-out cytochrome P450 (B-D, three mutants).

**Figure S14.** Extracted ion chromatograms obtained from the MYM culture extracts of *S. coelicolor* M1154 carrying the heterologous expression vector pYES-ACI-BGC2.28 (A) and *S. coelicolor* M1154 carrying the heterologous expression vector pYES-ACI-BGC2.28 with knocked-out cytochrome P450 (B). The extracted ion chromatograms show the [M+H]^+^ ions of acidomycin (C_9_H_15_NO_3_S, *m/z* 218.0845 ± 0.0050) and compounds with the sum formulae C_9_H_17_NO_3_S (*m/z* 220.1002 ± 0.0050), C_7_H_11_NO_3_S (*m/z* 190.0532 ± 0.0050), C_10_H_17_NO_4_S (*m/z* 248.0951 ± 0.0050), and C_10_H_17_NO_3_S (*m/z* 232.1002 ± 0.0050).

**Figure S15.** High resolution ESI-Qq-TOF MS/MS spectra of the [M+H]^+^ ions of stravidin S4 (A) and stravidin S5 (B).

References

1. Gómez-Escribano, J. P.; Bibb, M. J. Engineering Streptomyces coelicolor for heterologous expression of secondary metabolite gene clusters. Microb. Biotechnol. 2011, 4, 207–215.
2. Myronovskyi, M.; Rosenkranzer, B.; Nadmid, S.; Pujic, P.; Normand, P.; Luzhetskyy, A. Generation of a cluster-free Streptomyces albus chassis strain for improved heterologous expression of secondary metabolite clusters. *Metab. Eng.* **2018**, *49*, 316–324.
3. Flett, F.; Mersinias, V.; Smith, C. P. High efficiency intergeneric conjugal transfer of plasmid DNA from Escherichia coli to methyl DNA-restricting streptomycetes. *FEMS Microbiol. Lett.* **1997**, *155*, 223–229.
4. MacNeil DJ, Gewain KM, Ruby CL, Dezeny G, Gibbons PH, MacNeil T. Analysis of Streptomyces avermitilis genes required for avermectin biosynthesis utilizing a novel integration vector. Gene. 1992 Feb 1;111(1):61-8. doi: 10.1016/0378-1119(92)90603-m. PMID: 1547955.
5. Flett F, Mersinias V, Smith CP. High efficiency intergeneric conjugal transfer of plasmid DNA from Escherichia coli to methyl DNA-restricting streptomycetes. FEMS Microbiol Lett. 1997 Oct 15;155(2):223-9. doi: 10.1111/j.1574-6968.1997.tb13882.x. PMID: 9351205.
6. Brachmann, C. B.; Davies, A.; Cost, G. J.; Caputo, E.; Li, J.; Hieter, P.; Boeke, J. D. Designer deletion strains derived from Saccharomyces cerevisiae S288C: A useful set of strains and plasmids for PCR-mediated gene disruption and other applications. *Yeast* **1998**, *14*, 115–132.
7. Zotchev, S., K. Haugan, O. Sekurova, H. Sletta, T. E. Ellingsen, and S. Valla. 2000. 'Identification of a gene cluster for antibacterial polyketide-derived antibiotic biosynthesis in the nystatin producer Streptomyces noursei ATCC 11455', *Microbiology (Reading)*, 146 ( Pt 3): 611-19.
8. Xu, M., Wang, W., Waglechner, N. *et al.* GPAHex-A synthetic biology platform for Type IV–V glycopeptide antibiotic production and discovery. *Nat Commun* **11**, 5232 (2020). <https://doi.org/10.1038/s41467-020-19138-5>
9. Bilyk O, Sekurova ON, Zotchev SB, Luzhetskyy A. Cloning and Heterologous Expression of the Grecocycline Biosynthetic Gene Cluster. PLoS One. 2016 Jul 13;11(7):e0158682. doi: 10.1371/journal.pone.0158682. PMID: 27410036; PMCID: PMC4943663.
10. Tong, Y., C. M. Whitford, H. L. Robertsen, K. Blin, T. S. Jorgensen, A. K. Klitgaard, T. Gren, X. Jiang, T. Weber, and S. Y. Lee. 2019. 'Highly efficient DSB-free base editing for streptomycetes with CRISPR-BEST', *Proc Natl Acad Sci U S A*, 116: 20366-75.
